# Phytochemistry, Pharmacological Properties, and Recent Applications of *Ficus benghalensis* and *Ficus religiosa*

**DOI:** 10.3390/plants10122749

**Published:** 2021-12-14

**Authors:** Suganya Murugesu, Jinap Selamat, Vikneswari Perumal

**Affiliations:** 1 Institute of Tropical Agriculture and Food Security (ITAFoS), Universiti Putra Malaysia, Serdang 43400, Selangor, Malaysia; suganya@upm.edu.my; 2 Faculty of Food Science and Technology, Universiti Putra Malaysia, Serdang 43400, Selangor, Malaysia; 3 Faculty of Pharmacy & Health Sciences, University of Kuala Lumpur Royal College of Medicine Perak, Ipoh 30450, Perak, Malaysia; vikneswari@unikl.edu.my

**Keywords:** *Ficus benghalensis*, *Ficus religiosa*, phytoconstituents, bioactivity, medicinal uses

## Abstract

*Ficus* is one of the largest genera in the plant kingdom that belongs to the Moraceae family. This review aimed to summarize the medicinal uses, phytochemistry, and pharmacological actions of two major species from this genus, namely *Ficus benghalensis* and *Ficus religiosa*. These species can be found abundantly in most Asian countries, including Malaysia. The chemical analysis report has shown that *Ficus* species contained a wide range of phytoconstituents, including phenols, flavonoids, alkaloids, tannins, saponins, terpenoids, glycosides, sugar, protein, essential and volatile oils, and steroids. Existing studies on the pharmacological functions have revealed that the observed *Ficus* species possessed a broad range of biological properties, including antioxidants, antidiabetic, anti-inflammatory, anticancer, antitumor and antiproliferative, antimutagenic, antimicrobial, anti-helminthic, hepatoprotective, wound healing, anticoagulant, immunomodulatory activities, antistress, toxicity studies, and mosquitocidal effects. Apart from the plant parts and their extracts, the endophytes residing in these host plants were discussed as well. This study also includes the recent applications of the *Ficus* species and their plant parts, mainly in the nanotechnology field. Various search engines and databases were used to obtain the scientific findings, including Google Scholar, ScienceDirect, PMC, Research Gate, and Scopus. Overall, the review discusses the therapeutic potentials discovered in recent times and highlights the research gaps for prospective research work.

## 1. Introduction

*Ficus* have been known for their vast number of species, consisting of more than 800 species in the form of trees, vines, shrubs, epiphytes, and hemiphytes. *Ficus* genera belong to the Moraceae family of Urticales order under the classification of Dicotyledone and Spermatophyte phylum of the Plantae kingdom. There are more than 800 species of *Ficus* that have been discovered. *Ficus* plants are generally known as figs or fig trees. The genus is distributed in various regions across the tropical and sub-tropical areas, mainly in Asia, America, Australia, and Africa [1]. 

In India, some of the species are considered sacred, especially *Ficus benghalensis*, which is referred to as India’s National Tree that signifies spiritual knowledge and eternal life [2]. Some of the species are edible, while some are used as ornamental plants, especially *Ficus lyrata*, commonly known as the fiddle-leaf fig [3]. The common fig or *Ficus carica* Linnaeus is the most popular species of *Ficus*, known for its remarkable commercial importance, with multiple vernacular names such as Anjir (Hindi, Sanskrit, Malay, etc.), Fagari (Northern India), Thaphan (Burmese), Qua Va (Vietnamese), etc. [4]. Typically, all *Ficus* spp. have latex-like gummy material within the vasculatures that plays a role in the defense system and self-healing upon physical assaults. The latex is generally used as household detergents. Most species of this genus are characterized by their syconia, fleshy receptacles with ostioles at the apex that come in various shapes that develop into multiple fruits with duplets collection. Generally, the fresh fruits are sweet and juicy once ripe, containing thin and tender skin with a fleshy wall of different color variants such as red, pink, purple, etc., depending on the species [1]. 

Some of the species do not bear fruit, however they possess similar morphological features that are difficult to be distinguished from their species and variants. The veins’ ornamentation and traces in the lamina are added characteristics that aid in the species’ identification. Mainly, this genus grows with auxiliary root structures extended from their tree trunks or branches into the ground. As one of the diverse species, *Ficus* trees are some of the highest oxygen generators with the highest photosynthesis rate. The deeply lobed leaves were reported to contribute to the three major types of leaf minerals, namely calcium oxalates, amorphous calcium carbonate cystoliths, and silica phytoliths [5]. 

The genus displays various unique features that can be morphologically observed in its species. This review will highlight the two most abundant *Ficus* spp. available in Asian countries, especially in Malaysia, *Ficus benghalensis* and *Ficus religiosa.* Apart from those, *F. benghalensis* and *F. religiosa* are the two species that were explored extensively for their biological functions and bioactive compounds, besides their traditional applications. Thus, the review intends to update on the latest scientific findings of the two species, and thus indicate a gap in the research that may potentially be fulfilled in future studies.

## 2. Methods

### Literature Search

**Figure 1:**
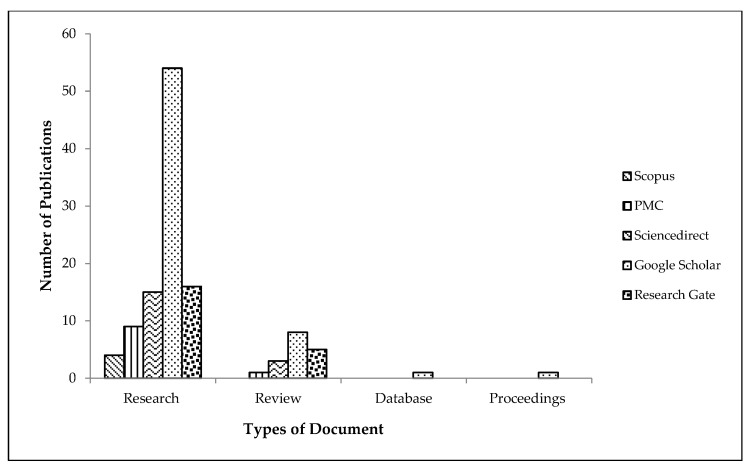
The sources and number of publications used.

Various search engines and databases were used to obtain the scientific findings on both the selected *Ficus* spp., including Google Scholar, ScienceDirect, PMC, Research Gate, and Scopus. Some of the terms or keywords used to search for potential publications included *Ficus* spp., *Ficus benghalensis*, *Ficus religiosa*, pharmacological activities, botany, nanotechnology, traditional uses, bioactivity, phytoconstituents, phytochemistry, etc. The total search yielded about 402 publications, that included research articles, review papers, book chapters, proceeding papers, and online notes. However, only research and review papers retrieved were used to present a comprehensive and updated review on the subject matter: about 38 papers of *Ficus benghalensis*, 40 of *Ficus religiosa*, 19 scientific findings on *Ficus* spp. and the rest are of nanotechnologyfollowed by publications on pharmacological activities and their definitions. One of the citations includes a plant database (Natural Resources Conservation Service). The rest of the documents were excluded considering the validity of the content with scientific proof. Figure 1 below shows the sources and number of publications used for this review. The chemical structures were generated using the ChemSketch software. 

## 3. Morphological Description and Traditional Uses

### 3.1. Ficus benghalensis

**Figure 2:**
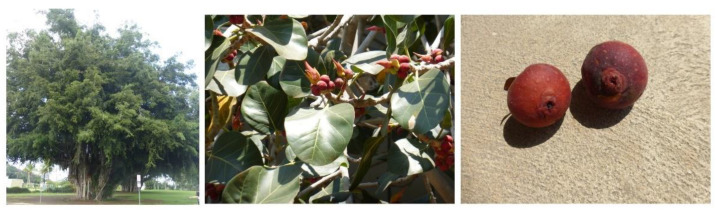
*Ficus benghalensis* tree, leaves, and fruits. Adapted from CalPhotos (2012) © 2021 Zoya Akulova [13].

Some of the common synonyms of the *F. benghalensis* plant include the Banyan tree, Indian fig, and Nyagrodha. The sacred Banyan tree of India, that comes from the *Urostigma* subgenus, branches into a great number of shoots that take roots and become a new trunk, and thus are grown mainly in gardens and on roadsides for shade. The tree is known to be epiphytic when young, with petioles of 1.25 to 5 cm length, ovate lamina sessile, and reddish hypanthodia upon maturation. The female flowers are pedicellate, elongated with about 3 to 5 mm in length. However, male flowers are absent in the same stalk [5]. Typically, the plant parts are used in the preparation of Ayurvedic remedies in India [6]. The plant parts are known for their astringency due to the presence of tannins. Despite its astringency, it is also known for its cooling effect, as alterative and demulcent. *F. benghalensis* (Figure 2) root and stem bark are typically prepared in decoction form to treat a variety of conditions, such as dysentery, diarrhea, skin disorders, inflammation, and diabetes [6,7]. Meanwhile, the leaf portion is consumed to boost the immune system, and as a remedy for leucorrhea and other vaginal discharges [7,8]. Latex produced by this plant is utilized in promoting conception, as a blood purifier in urinary and urinogenital disorders [9,10]. The seeds of *F. benghalensis* are prescribed as a dietary supplement for peptic ulcers by traditional medicine practitioners [11]. However, the fleshy fruit of *F. benghalensis* is not edible for humans due to its laxative nature [12].

### 3.2. Ficus religiosa

**Figure 3:**
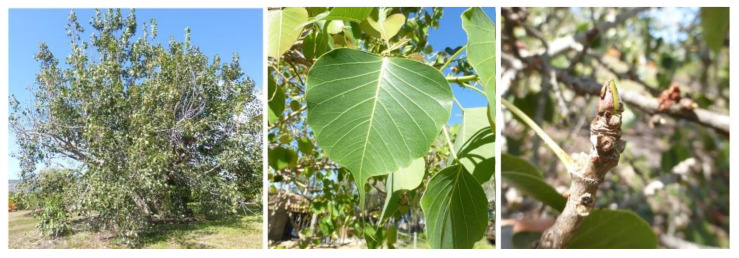
*Ficus religiosa* tree, leaves, and fruits. Adapted from CalPhotos (2012) © 2021 Zoya Akulova [13].

*Ficus religiosa* is one of the widely planted species of *Ficus* in the tropics, with various traditional applications. It belongs to the subgenus of *Urostigma* and is locally known as the Peepal tree (synonym: Pimpala). It is a large tree (Figure 3) and epiphytic when young, containing petioles of 5 to 10 cm in length, aspen-like lamina, sessile, and paired hypanthodia, with no male flowers and pedicellate or sessile [5]. Its bitter-sweet and acrid nature is the reason for its use as an astringent, refrigerant, purgative, aphrodisiac, and laxative [9]. The root bark is often used to treat stomatitis, ulcers, and other inflammatory conditions such as gout [14]. The laxative young fruit is known to promote digestion and treat vomiting [15]. The ripe fruit of this species is edible and commonly used in food preparation. The fruits are rich in antioxidants, minerals, and vitamins [16]. The leaves are usually applied to wounds, skin diseases, and scabies [15]. The leaves are also prepared as a tonic for ulcers and constipation [14]. The young shoots are purgative and are thus used in the treatment of various conditions, including urinary vaginal discharge, asthma, cracked foot, toothache, snake bite, pimples, otitis, sores, etc. [9].

## 4. Phytochemical Constituents

**Figure 4:**
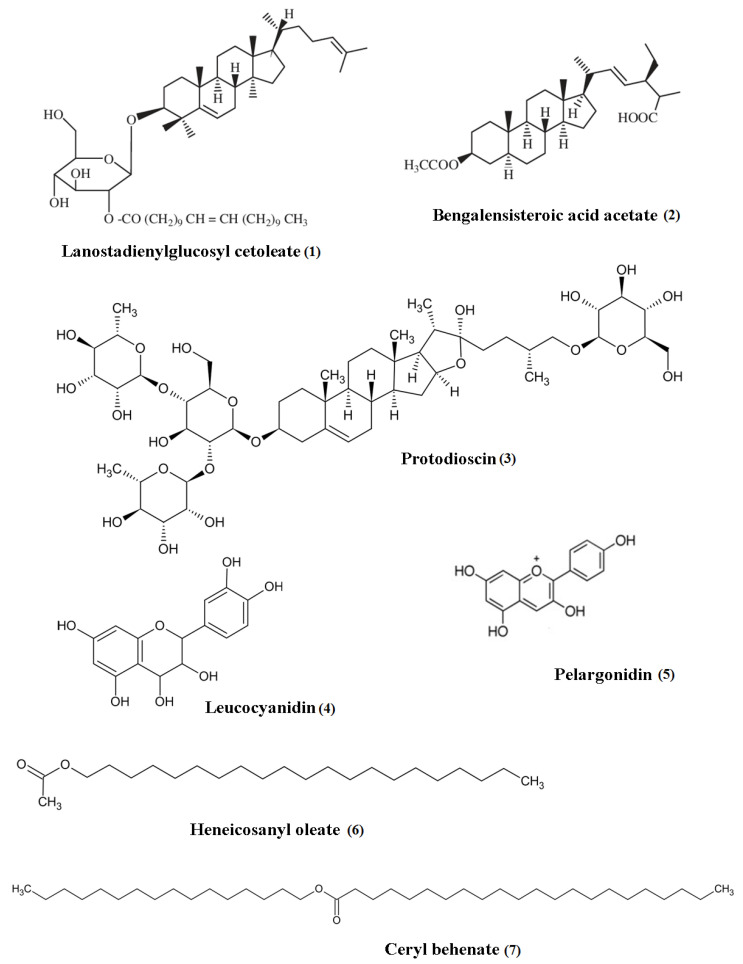
Distinctive compounds present in *Ficus benghalensis* (**1**–**5**) and *Ficus religiosa* (**4**, **6**, **7**).

Plants are the source of various phytochemical constituents that are functional as a remedy for health defects that occur in humans, and the diversity of the plant metabolites benefits humans in treating those conditions [1,2]. *Ficus* species is one of the largest genera of the plant kingdom, with promising phytoconstituents from various classes of compounds, including phenols, flavonoids, sterols, alkaloids, tannins, saponin, terpenoids, etc. Table 1 displays the phytoconstituents of the discussed *Ficus* species and the plant parts containing them. Some of the distinct compounds identified from both species are displayed in Figure 4. 

**Figure 5:**
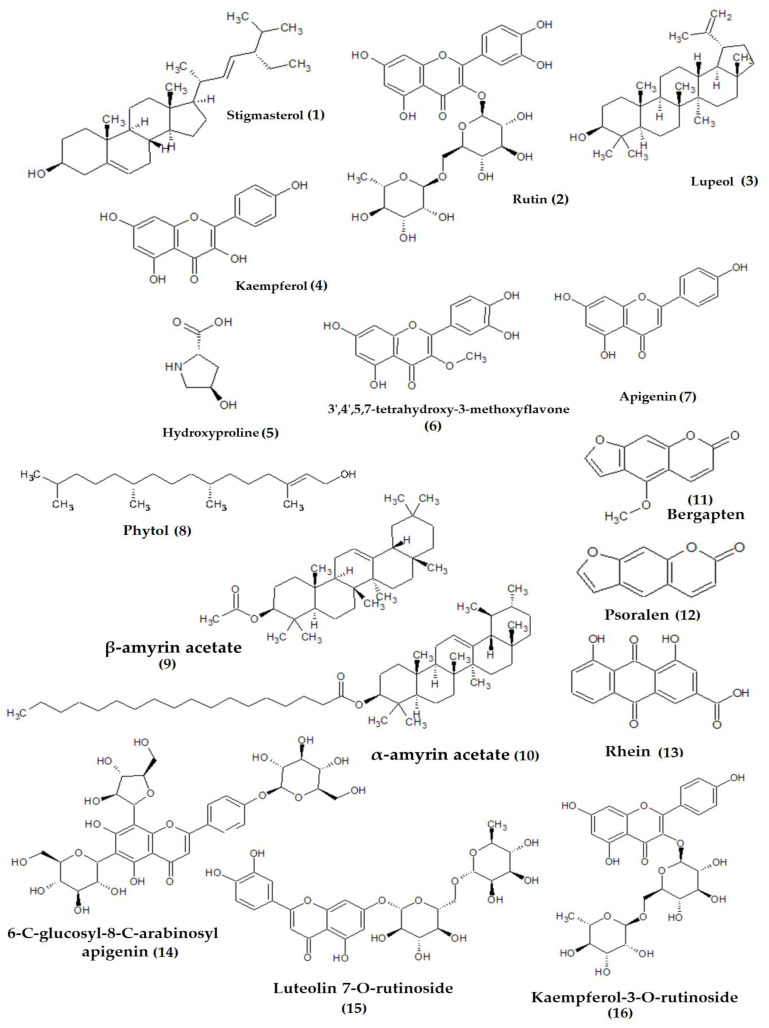
Chemical structure of bioactive compounds reported from *Ficus benghalensis* (**2**–**5**, **8**–**10**, **11**–**13**) and *Ficus religiosa* (**1**, **6**–**10**, **11**, **14**–**16**).

The leaves and bark of *F. benghalensis* are rich in flavonoids, phenols, terpenoids, and terpenes [17,18,19,20]. Besides that, the leaves also contain quinone and furanocoumarin derivatives, namely rhein, psoralen, and bergapten (Figure 5) [17]. The root extract consists of sterols and organic and fatty acids, while the fruit was reported to be rich in fatty acids [21]. As for *F. religiosa*, the fruits mainly contain terpenes [23]. The leaves are rich in amino and fatty acids, as well as terpenoids [23,24]. The latex of *F. religiosa* contains serine protease named Religiosin B and C [30,31]. 

Apart from these plant parts, *Ficus* species are known for their parasitic host effect in the ecosystem for various endophytic fungi. These fungi inhabit the host plant by living in their tissues, exhibiting a mutual relationship without causing any disease to the host. The parasitic fungi play a role in the host plants’ defense mechanism by producing bioactive secondary metabolites. Some of these metabolites possess similar functions as the ones produced by the plants [32]. Some of the fungi were analyzed for their metabolites with medicinal effects, which will also be highlighted (Table 2) in this review. Apart from the fungi, *Bacillus subtilis*, a catalase-positive bacterium, was identified and isolated from the aerial root of *F. benghalensis*. Two anti-fungal compounds (surfactins and iturins) produced by the bacterium were isolated (Table 2) [33]. The screening analysis has revealed the presence of phenols, flavonoids, alkaloids, terpenes, and terpenoids from the fungi extracts [32,34,35].

## 5. Pharmacological Actions

**Figure 6:**
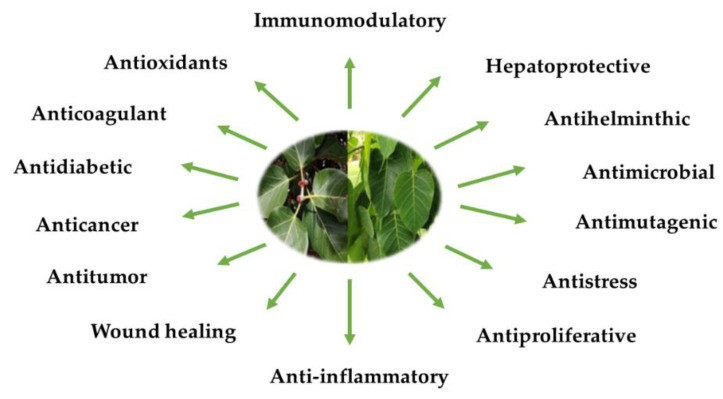
Pharmacological activities of *Ficus benghalensis* and *Ficus religiosa*.

*Ficus* species have been vastly studied for multiple pharmacological effects (Figure 6), as discussed below. All the plant organs, including leaves, stem bark, root, latex, and fruits, were investigated for their potential bioactivities. Some of the bioactivities researched include antioxidants, antidiabetic, anti-inflammatory, anticancer, antitumor and antiproliferative, antimutagenic, antimicrobial, anti-helminthic, hepatoprotective, wound healing, anticoagulant, immunomodulatory activities, antistress, and toxicity studies. The plants are also utilized as insect repellents.

### 5.1. Antioxidants

Plants are typically rich in diverse antioxidant compounds, which contribute to most of their biological effects in the human system. Antioxidants can inhibit oxidation via termination of radical reaction by donating at least one hydrogen atom to the free radical or by preventing the initiation of radical chain reaction via a substitutive reaction. Therefore, cells must preserve the levels of antioxidants through dietary and de novo synthesis [36,37,38].

The presence of antioxidants has been studied extensively in *Ficus* species using various antioxidant assays. *F. benghalensis* root’s aqueous extract was reported to possess the highest scavenging activity and reducing power compared to its methanolic and ethanolic extracts [21]. The phytochemical screening of the *F. benghalensis* root revealed the presence of steroids, flavonoids, tannins, phenolic compounds, and anthraquinone glycoside as its major constituents [39]. Meanwhile, the aqueous extract of *F. benghalensis* stem bark displayed a significant inhibition (IC_50_ = 80.24 μg/mL) compared to the standard reference used, tetraethoxypropane. The measurement was performed based on the thiobarbituric acid reactive substances (TBARS) value that measures the lipid peroxide generation as tested on microsomal lipid peroxidation [40]. Besides that, *F. benghalensis* latex methanolic extract showed potential scavenging activity of 1-1-diphenyl-2-picrylhydrazyl (DPPH), ferric chloride (FeCl_3_) reducing antioxidant power (FRAP), and phosphomolybdenum (IC_50_ = 28.63, 49.82, and 31.84 µg/mL, respectively) as compared to the reference compounds (ascorbic acid and Trolox). The activity is reportedly due to abundance in flavonoids and phenolics, corresponding to their preliminary phytochemical screening [41].

Apart from those plant parts, the leaves’ extracts (hydroethanolic and hexane) of *F. benghalensis* have also shown potential DPPH scavenging activity (IC_50_ = 32.3 and 28.2 µg/mL) that are comparable with the reference compounds used (ascorbic acid 11.5 µg/mL and quercetin 15.4 µg/mL) [42]. In addition, the ethanolic extract of *F. benghalensis* seed was reported to possess potential antioxidant capacity with a high abundance in tannins, flavonoids, and phenolics content [10]. *F. benghalensis* plant parts were reported to contain carbohydrates, phenolic compounds, oil, fats, saponins, flavonoids, alkaloids, proteins, and tannins as major constituents that contribute to their antioxidant capacity, which subsequently influences the pharmacological effects [17].

*F. religiosa*, the sacred fig, is known to exhibit potent antioxidant capacity contributed by phenolic and flavonoids (1032 mg GAE/g extract and 63.31 mg QE/g extract, respectively) in its fruit [43]. Meanwhile, the *n*-hexane, dichloromethane, and ethyl acetate fractions of the *F. religiosa* stem bark were reported to possess high scavenging activity with >90% inhibition compared to the crude methanol and *n*-butanol fraction [44]. Similarly, Ashraf et al. [45] reported that the ethanolic extract of the *F. religiosa* exhibits strong scavenging activity compared to other extracts analyzed. Added to this, the methanolic extract of *F. religiosa* bark displayed a significant antioxidant activity with the IC_50_ value of 48 μg/mL [46]. Another study was conducted using the combined methanolic extracts of *F. religiosa* and *F. benghalensis* leaves, which exhibited significantly good scavenging activity of DPPH with an inhibition percentage slightly higher than the standard (ascorbic acid) used. The study also displayed the hydrogen peroxide scavenging ability of the extract with the IC_50_ value of 49.85 µg/mL, much lower compared to the standard (80.09 µg/mL). The activity may be contributed by the extract’s constituents identified via GC/MS by the same team, reportedly comprised of amine, aldehyde, and aromatic groups, with squalene and amyrin acetate as the major compounds [47]. 

One of the recent studies by Jayant and Vijayakumar [32] has shown the pharmacological activity of the secondary metabolites extracted from the endophytic fungi isolated from *F. religiosa*. Among the ten fungi isolated and tested, *Curvularia lunata* displayed the highest radical scavenging activity with the lowest IC_50_ value of 0.42 mg/mL. 

### 5.2. Antidiabetic

The management of diabetes involves multiple mechanisms that biologically affect the pathogenesis of the disorder. These include the inhibition of glucose hydrolyzing enzymes to reduce the postprandial glucose level [48,49,50]. Both the *Ficus* species observed in this study have been explored for their antidiabetic effects using an in vitro assay and various animal models. 

The in vitro study analyzing the carbohydrate hydrolyzing enzyme inhibition activity using *F. benghalensis* bark powder extract demonstrated potential activity. The aqueous extract of the bark powder measured IC_50_ values of 77 and 141 µg/mL, against both α-glucosidase and sucrose enzymes, respectively [51]. Meanwhile, in an in vivo study using the ethanolic leaves’ extract (200 mg/kg, 400 mg/kg body weight) from *F. benghalensis* on alloxan-induced diabetic albino rats, the extract reportedly reduced the triglycerides, cholesterol, and glucose levels, signifying the traditional use of plant leaves as antidiabetic agents [52]. In another study, oral administration of *F. benghalensis* bark extract was reported to lower blood glucose in streptozotocin (STZ)-induced diabetic rats through the stimulation of insulin secretion from beta cells of Islets of Langerhans [53]. Apart from that, an in silico study has revealed the potential α-glucosidase inhibitors reported in *F. benghalensis* that can inhibit the aldose reductase enzyme. The aldose reductase is the enzyme involved in the glucose metabolism pathway that is crucial in the management of diabetes mellitus. The three flavonoids (apigenin, 3,4’,5,7-tetrahydroxy-3’-methoxy flavone, and kaempferol) investigated showed a high affinity towards the enzyme observed and were predicted to modulate most protein molecules via the p53 signaling pathway [54].

The aqueous extract of *F. religiosa* bark has successfully reduced the blood glucose level in a STZ-induced diabetic rat model in a dose-dependent manner. The extract also improved the insulin level and glycogen content in the liver and skeletal muscle. Besides that, a significant reduction in serum triglyceride and total cholesterol along with reduced lipid peroxidation were observed upon treatment. The extract displayed blood glucose normalization within four hours of treatment in all treated groups [55]. Another study using the methanolic extract of the *F. religiosa* bark reported a strong antidiabetic activity with the IC_50_ value of 83.72 μg/mL, displaying potential antihyperglycemic activity [56]. 

The bioactivity determined in the metabolites extracted from the endophytic fungus (*Curvularia lunata*) isolated from *F. religiosa* exhibited good α-amylase inhibition activity (80%) [32]. Another similar study isolated nine endophytic fungi which were then colonized and extracted using three different solvents: petroleum ether, diethyl ether, and ethyl acetate. All the recovered extracts were analyzed via α-amylase and α-glucosidase inhibition assays, followed by a glucose diffusion assay. The isolated fungi that displayed good inhibition were suggested to be *Aspergillus* species based on their microscopic view. The petroleum ether extract of the fungi showed 91% inhibition against α-amylase and average α-glucosidase inhibition (42%). The same extract managed to prevent glucose efflux via maximum inhibition of the glucose movement outside the membrane [57]. Another species of fungus isolated and extracted was the *Aspergillus* species, which yields two naphthoquinones, namely naphthoquinone antibiotic herbarin and herbaridine A. The first compound was reported to induce glucose uptake in rat skeletal muscles in the presence of insulin at a low concentration (EC_50_: 0.8 µM) compared to the standard, Rosiglitazone (EC_50_: 3.0 µM), thus indicating its potential to be developed as an antidiabetic drug [35].

### 5.3. Anti-Inflammatory

Inflammation is a defense mechanism towards harmful stimuli, such as pathogens, injured or damaged cells, irradiation, or toxic compounds, thus initiating the healing process [58,59]. Inflammation is often associated with major diseases including cancer, diabetes, heart diseases, etc., via multiple mechanisms. Various phytoconstituents have the potential to interfere with the mechanisms and combat a series of inflammation [60]. One of the mechanisms that involves inflammation is an injury to the skin and other soft tissues, and the wound healing process. An inflammatory response occurs upon injury that subsequently induces the cells underlying the dermis layer to increase collagen production, followed by epithelial tissue regeneration in response to healing [61]. 

*F. benghalensis* is one of the *Ficus* spp. that is widely used for wound healing. The healing property of this species was investigated using its leaves’ ethanolic extract on excision and incision wound models [62]. The extract (200 mg/kg dose) had effectively accelerated wound healing through a decreased epithelization period, increased wound contraction rate, and skin breaking tensile, along with the absence of mortality and toxicity signs at a 2000 mg/kg dose. The root’s ethanolic extract has also exhibited wound healing properties. The extract has significantly increased the closure of the excision wound on the rat by enhancing the epithelization. The extract also increased the granuloma tissue-breaking strength, indicating progressive healing [63].

Meanwhile, another study investigated the antiulcer activity of *F. benghalensis* leaves’ methanolic extract using the gastric ulcer rat model at the dosage of 250 and 500 mg/kg via oral administration [14]. The extract was measured to effectively reduce the ulcer index in the aspirin-induced gastric ulcer model, thus indicating its anti-ulcerogenic potential. It is suggested that the gastro-protective effect of the extract is exhibited through the action against the 5-lipoxygenase pathway induced by aspirin. The disruption of this pathway stimulates the prostaglandin synthesis, which eventually protects the gastric mucosa [14]. Similarly, another antiulcer study used three different animal model to test the *F. benghalensis* leaves’ methanolic (50%) extract was conducted. The first model is of pylorus ligation and aspirin-induced gastric ulcer where 100, 200 and 400 mg/kg were orally administered for three days. The treatment showed reduction of damage in the mucosa with reduced ulcer indices and increased protection percentage in dose-dependent manner. The second model is acetic acid- induced gastric ulcer with ten days intervention while the third model is of ethanol-hydrochloric acid-induced gastric ulcer for eight days treatment with the same dosages. Both models displayed potential antiulcer activity with improved damage in the mucosa and ulcer indices and protection percentage [64].

Arthritis is a chronic inflammatory disorder that occurs in the joints and may progress with age [60]. The aqueous and ethanol extracts of *F. benghalensis* bark were shown to exhibit anti-arthritic activity, that was displayed via stabilization of protein denaturation in a dose-dependent manner [65]. The bark extracts showed the presence of a significant amount of terpenoids, saponin, flavonoids, and phenol [65]. *F. religiosa* leaves’ ethanolic extract (200–400 mg/kg) was analyzed for its anti-arthritic effect in Freund’s complete adjuvant-induced arthritis rat. The treatment was conducted for 21 days and multiple parameters were measured, including body weight, arthritic score, ankle diameter, and paw volume, which were normalized after the treatment with protective effects exerted by the extract on the primary and secondary lesions [24]. 

Pain is a sensation caused by any intense or damaging stimulation that occurs in response to tissue damage and inflammation. Pain can be blocked using anti-inflammatory or analgesic drugs [66]. The analgesic effect of *F. benghalensis* aqueous root extract (100 and 200 mg/kg) was tested using Swiss albino mice. Some of the tests carried out include the hot-plate, tail-flick, and writhing tests. A significant observation was recorded in the writhing test upon treatment with the root extract, indicating the potential analgesic effect of the plant root [66].

The anti-inflammatory potential of another *Ficus* spp. was investigated using its latex on cisplatin-induced liver injury. An in vivo study carried out by Yadav and Srivastava [67] has demonstrated the hepato-curative and nephroprotective effects of *F. religiosa* latex (defatted) methanolic extract. The latex-treated group with cisplatin-induced liver injury has shown protective and curative symptoms upon treatment with 200 and 300 mg/kg, with an observable reduction in the hyaline droplets, tubular dilation, and recovery. The negative impacts caused by cisplatin were reversed upon treatment with the latex. The elevated serum urea and creatinine, as well as lipid peroxidation, were normalized. Besides that, normalization of the renal biomarkers, namely glutathione (GSH), superoxide dismutase (SOD), catalase (CAT), and ATPase (Na^+^/K^+^, Ca^2+^, and Mg^2+^), were observed. They have concluded that the antioxidant content in the latex has played an important role in the protective and curative effects of the liver injury and renal profile. 

The anti-inflammatory activity of two naphthoquinones extracted from *Dendryphion nanum* (Nees), an endophytic fungus isolated from *F. religiosa* leaves, was reported by Mishra et al. [35]. Compound 1 was identified as naphthoquinone antibiotic herbarin, which effectively inhibits the TNF-α (IC_50_: 0.06 µM) and IL-6 (IC_50_: 0.01 µM) cytokines’ production in the LPS-induced human mononuclear cell line, which was similar to that of the standard drug used in the assay, Dexamethasone. Meanwhile, the other compound isolated and identified as herbaridine A was found to be inactive against the same activity.

The wound healing properties of aqueous extracts obtained from *F. religiosa* bark, leaves, and aerial roots were tested using cell culture, real-time polymerase chain reaction (PCR), astringent activity, and the wound healing assay. Downregulation of the metalloproteinase-1 (MMP) matrix was observed in the PCR analysis using the bark and aerial root extracts. The bark and leaf extracts were found to enhance the wound healing area [46]. 

Recently, the bark extract and ash were tested on the burned wound on Sprague Dawley rats. Different extracts of ointment were formulated for topical application, of which the ash and the aqueous extract formulation were found to be effective in healing the burned wound. The methanolic and chloroform extract formulation was found to be the least reactive. The 100% wound contraction was observed after 15 days of treatment in both the bark ash and aqueous extract ointments, leading to a lower wound closure time, indicating healing. A progressive re-epithelization, formation of granulation tissues, followed by cellular proliferation was observed in the plant-treated wound [68].

### 5.4. Antitumor, Antimitotic, and Antiproliferative 

Tumor formation is an early stage of cancer evolution. However, a tumor can be benign, forming a mass that enlarges, is localized, and is treatable. A malignant tumor is referred to as cancer that can be deadly. The continual proliferation of the malignant cells is the mechanism by which cancer cells metastasize in no time, and it leads to mortality if not detected [69]. Therefore, finding novel antitumor, antimitotic, and antiproliferative agents would be of help to the communities that are uncertain about the mass formation and effects. *Ficus* spp. has been investigated for their antitumor activity as well. *F. benghalensis* stem bark butanol fraction was shown to possess the strongest antiproliferative activity with the lowest viability of 8% at 4 mg/mL [70]. The same study has investigated the antimitotic action of the crude methanol and fractions from stem bark. Similarly, the *n*-butanol fraction showed the strongest antimitotic activity, with a mitotic index of 28% at the same concentration. The mechanism involved the induction of chromosomal and mitotic aberrations, which can be observed through the accumulation of prophases, sticky chromosomes at metaphase, followed by spindle disturbance at prophase and anaphase bridges.

### 5.5. Anticancer 

The search for an anticancer plant and its constituents is ongoing, extensively applying various cell lines. 

*F. benghalensis* latex extracted in various solvents was screened for its antiproliferative activity on multiple cell lines, including colorectal, human breast, neuroblastoma, and lymphocytes [71]. Ethanol extract has shown promising results against colorectal and neuroblastoma cells, while ethyl acetate extract against the human breast cell lines. Both the extracts were also discovered to exert lesser toxic effects on peripheral blood lymphocytes [72]. Another study investigated the ethyl acetate extract of *F. benghalensis* aerial roots to assess its anticancer activity on lung cancer (A549), breast cancer (MDA-MB-231), and cervical cancer (Hela) cell lines. The extract showed potential activity with the IC_50_ values of 17.81, 97.89, and 49.27 µg/mL, respectively [73].

Besides that, *F. religiosa* plant parts were also investigated for their anticancer activity using various cancer cell lines. The aqueous extract of *F. religiosa* bark reduced the growth of the cervical cancer cell lines (SiHa and HeLa). The mechanism of action involves upregulating the expression of p53, p21, and pRb proteins. This was followed by downregulation of the phospho Rb (ppRb) protein expression, that subsequently terminates the cell cycle progression at the G1/S phase in SiHa. On the other hand, the extract was found to induce apoptosis in HeLa by increasing the intracellular Ca^2+^ level, which results in the loss of mitochondrial membrane potential. It also promoted the release of cytochrome-c and upregulated the caspase-3 expression. Overall, the mechanism of action involved the downregulation of MMP-2 and Her-2, as well as viral oncoproteins E6 and E7 expression in both the cell lines [74]. The results are in accordance with those reported by El-Hawary et al. [75] and Kumaresan et al. [48] on the same cell line, however, using the methanolic extract. 

Gulecha and Sivakumar [76] have reported the *F. religiosa* leaves’ extract and fraction to be effective in attenuating the viable breast cancer cells. The extract caused apoptosis in breast cancer cells (MCF-7) via multiple cellular signaling mechanisms. Cell cycle analysis showed that cell arrest occurs in the G1 phase. The extract was observed to induce chromatin condensation, resulting in the apoptotic population being increased, as well as causing the loss of mitochondrial membrane potential in the cells. Subsequently, the extract upregulated caspase 9 expression and accelerated mitochondria-mediated cell death.

Recently, *F. religiosa* latex’s ethanol extract was tested against three different cell lines, namely human neuroblastoma IMR 32, human colorectal HCT 116, and human breast adenocarcinoma MDA MB 231. The extract was found to be toxic against all the cell lines observed in this study, with the lowest inhibitory value of 4.8 μg/mL measured against the neuroblastoma cell line. The mechanism of action observed via cell cycle analysis revealed cell arrest and accumulation at the G1 phase in both adenocarcinoma and colorectal cell lines, whereas similar actions were observed in the G2/M phase of IMR 32 cells. The extract induced apoptosis action, which indicates the upregulation of pro-apoptotic (caspase-3 and p53) and downregulation of anti-apoptotic (Bcl-2, AKT) genes [77]. A similar study using the methanolic bark extract (91 µg/mL) on human breast adenocarcinoma was conducted by Shankar et al. [78]. The study revealed that maximum cell death was observed in the treated cells; in contrast, minimal apoptosis or necrosis was observed in the non-cancerous cell line (HEK 293 T) tested, simultaneously. This indicates the selectivity of the potential compound(s) in targeting cancerous cells. The extract stimulated early apoptosis and apoptosis in cells, with about 86.3% apoptotic cells in the G0/G1 population. The gene expression indicates that the mechanisms involved were upregulation of BAX and proteolytic cleavage of PARP-1, and downregulated Bcl-2 genes. The anticancer activity of the bark extract is reportedly due to the presence of potential metabolites, which include rutin, 3-caffeoylquinic acid, luteolin 7-O-rutinoside, 6-C-glucosyl-8-C-arabinosylapigenin, and kaempferol-3-O-rutinoside (Figure 5), detected from the UPLC-MS analysis.

### 5.6. Antimutagenic

Mutagenicity is described as the induction of permanent transmissible changes in the structure or quantity of the genetic material of cells or organisms that consequently lead to certain diseases. Antimutagenic agents can prevent this in the first place before the phenomenon could take place by inhibiting and suppressing the known mutagens [79]. Some of the plant metabolites can act as antimutagens that can prevent mutagenicity in certain cells and their genetic materials. *F. benghalensis* aqueous stem bark demonstrated a potential antimutagenic effect on *Salmonella typhimurium* TA100 strains with the IC_50_ value of 70.24 mg/mL [40].

### 5.7. Antimicrobial

Microbes are microorganisms, including bacteria, viruses, fungi, and others, that may cause infectious and deadly diseases if acquired into any biological system. An antimicrobial agent refers to natural or synthetic components that can kill or inhibit the growth of those microorganisms. The increased multi-drug resistance organisms have increased the search for novel antimicrobial agents from the natural source, plants [80].

The ethanolic extract of *F. benghalensis* root showed good growth inhibition with increased concentration (25, 50, and 75 mg/mL) in three strains of bacteria (*Staphylococcus aureus, Escherichia coli*, and *Klebsiella pneumonia*), as reported by Murti and Kumar [19]. The extract has effectively inhibited the *S. aureus, E. coli*, and *K. pneumonia* with the diameter of inhibition zones 30, 24, and 22 mm respectively, at its highest concentration compared to the standard drug (Ampicillin) used, with 40, 35, and 35 mm inhibition zones. A similar study using methanol and ethanol extracts of *F. bengalensis* aerial root suggested that the extract is more sensitive against *Vibrio anguillarum* and *Enterococcus faecalis* with the diameter of the zone of inhibition of more than 20 mm [21]. Previous studies using alcohol extract of *F. bengalensis* plant parts (leaf, root, and fruit) against *S. aureus*, *E. coli*, *Pseudomonas protobacteria*, and *Bacillus cereus* showed moderate activity [81].

In recent times, *F. bengalensis* leaves’ aqueous extract showed moderate activity upon investigation for its antiviral activity, which was screened using both cell-free and cell-associated assays against primary isolates of Human Immunodeficiency Virus (HIV), HIV-1UG070, and HIV-1VB59 in TZM-bl and PM1 cell lines [82]. 

The Gram-negative *K. pneumonia* showed more sensitivity towards the ethanolic extract of *F. religiosa* fruits’ extract (15 mg/mL) than *S. epidermidis* with the inhibition zones of 21 and 19 mm, respectively. The other two strains of bacteria were less vulnerable towards the extract, even at 30 mg/mL [83]. The antifungal activity was reported on *F. religiosa* bark ethanol extract, which exhibited moderate activity against *Candida albicans*, an opportunistic pathogenic yeast [84].

### 5.8. Anti-Helminthic Activity

Helminth infections are parasitic infestations in the human system that affect the world’s populations, mostly in developing countries with poor sanitation. Humans can become infected through ingestion or skin penetration. The parasite could deprive the host of food, resulting in blood loss, organ and intestinal damage, or lymphatic obstruction, that eventually causes death. Anti-helminthic drugs react by killing or expelling the infesting helminth from the host body. Generally, helminthiasis is not fatal, however, it can cause morbidity. The medications may cause some side effects, including fever, dizziness, nausea, severe allergies, and headache [85,86]. In most developing countries, medicinal plants are used as anti-helminthic agents. *F. bengalensis* aerial roots’ methanol and leaves’ ethanol extract exhibit the same action against the same species analyzed [87,88,89]. Meanwhile, for the latex of *F. bengalensis* and *F. religiosa*, the latter exhibits the most effective activity with a much faster time to cause paralysis in the earthworms analyzed, eventually leading to death [90]. Both the plants can be utilized as anti-helminthic in communities with less access to proper medications. 

### 5.9. Hepatoprotective 

The liver is the key organ that is responsible for regulating metabolism, secretion, storage, and detoxifying activity in the body, which will distort those functions if the organ is affected in any way [91]. Therefore, it is crucial to ensure the liver is protected from various kinds of toxic components acquired via food, synthetic medicines, and other factors. 

*F. bengalensis* latex was orally administered to CCl_4_-induced hepatotoxicity in albino rats and paracetamol-induced hepatic damage in rats. The latex treatment showed improvement to liver function with a significant reduction in the serum glutamate oxaloacetate transaminase (SGOT), serum glutamate pyruvate transaminase (SGPT), and bilirubin and alkaline phosphate (ALP) levels, and improvement in the total protein level [92]. The investigation of the hepatoprotective effect of the *F. bengalensis* fruit extract on goat liver assessed through catalase activity showed that the ethanol extract of the fruit at the dose of 50 mg/kg could significantly reduce the hepatoxicity effect against Silymarin [93].

Recently, the fruit of *F. benghalensis* was evaluated for its hepatoprotective effects via in vitro assays. The fruit was extracted using different solvent systems, namely ethanol, water, chloroform, ethyl acetate, and petroleum ether. The hepatotoxicity condition was induced using carbon tetrachloride, acetaminophen, and erythromycin in the liver extracted from goat, *Capra Capra*, and treated with 100, 250, and 500 mg/kg of the fruit extracts. Of all the extracts tested, the ethanol extract was found to be most effective in reducing the hepatotoxic activity at the dose of 500 mg/kg of fruit extract against Silymarin, the control drug [94]. This finding is supported by an in vivo study conducted recently using the fruit’s ethanolic extract (500 mg/kg) against perchloromethane-induced toxic hepatitis in New Zealand albino rats, indicating the hepatoprotective action of *F. benghalensis* fruit. The elevated liver biomarkers (alanine aminotransferase (ALT), aspartate aminotransferase (AST), total serum bilirubin, and malondialdehyde) were reduced upon administration of the fruit extract [95]. The study suggested that the diminution of lipid peroxides potentially by the antioxidant compounds in the fruit may have contributed to lipid-protective action. The fruit is known to be rich in coumarins, that reportedly possess hepatoprotective activity.

Oral administration of *F. religiosa* leaves’ extracts to CCl_4_-induced hepatotoxicity in albino rats and paracetamol-induced hepatic damage in rats displayed a significant reduction in the serum aspartate aminotransferase (AST) and alanine aminotransferase (ALT), which will be elevated in a liver-damaging phenomenon. The *F. religiosa* leaves’ extract was found to reduce those biochemical markers, thus improving liver function [96].

### 5.10. Anti-Aging

Wound healing is the skin repair process that occurs naturally after damage, which is important in restoring the tissue’s normal function [97]. *F. bengalensis* and *F. religiosa* are traditionally used in wound healing [98]. The ethanolic extract of the *F. benghalensis* root has significantly improved collagen synthesis. It showed high content of hydroxyproline that probably stimulates collagen synthesis [97]. Meanwhile, the aqueous extracts of *F. religiosa* bark and leaves were found to have a significant skin-tightening effect [46]. 

### 5.11. Anticoagulant Activity 

Anticoagulant activity is the process of hindering thrombus formation or blood clotting, thus preventing coagulation or reducing the formation of new blood clots, especially in patients with a high risk of having a stroke. Anticoagulant agents can lead to gastrointestinal problems in the long run [99]. Therefore, the findings on the potential anticoagulant agents from medicinal plants are encouraged. Evaluation of *F. benghalensis* and *F. religiosa* leaves’ methanolic extract in human plasma showed significant activity, with prothrombin time (PT) ranging from 17.7 to 26.7 s and activated partial thromboplastin time (APTT) varying from 47.7 to 72.3 s. *F. religiosa* extract showed better activity than the other species [99].

### 5.12. Immunomodulatory Activity

Modulation of the immune system or immunomodulatory activity is an important mechanism that regulates the activity of the immune system by stimulating or suppressing its functions [100]. *F. benghalensis* leaves’ hydroethanolic extract has displayed a potential immunomodulatory activity by stimulating the neutrophils to phagocytic action significantly, by 45%. The butanol fraction obtained from the crude hydroethanolic extract showed the highest results, with 89% stimulation of the phagocytic activity. Furthermore, the crude extract stimulated the phagocytosis of the killed *Candida albicans* at 1000 mg/mL, which is comparable to the pooled serum activity [42]. The *F. benghalensis* aerial root’s methanolic extract showed an elevated response for the plaque-forming response and quantitative hemolysis assay. The extract also stimulated the production of a circulating antibody titer in response to sheep red blood cells. The extract was found to increase the delayed-type hypersensitivity response by facilitation of the footpad thickness response and improved lymphocytes and rosettes formation. The results are comparable to that of the standard, Levamisole, and the extract exhibited potential immunomodulatory effects via a specific and non-specific immune response [101].

### 5.13. Antistress 

Stress is an emotional and physical tension that causes psychological and physiological disturbance when ones’ emotions are challenged or when a certain demand in life is not fulfilled. In this era of a demanding world, life has been stressful for many individuals, which has led to increased cases of depression in current times. Humans are dwelling in stress and intolerance on a daily basis. The psychological and physiological effects from natural sources will be of help, rather than medicines. *F. benghalensis* has scientifically shown potential antistress activity [102,103]. The fruits’ methanolic extract were investigated using the anoxia stress tolerance test, swimming endurance test, and immobilization stress animal models, exhibiting significant antistress results at the minimal dosage tested (125, 250, and 500 mg/kg) [102]. The bark (methanol extract) was tested using acetylcholinesterase inhibitory activity against SHSY5Y cell lines, measuring a 228.3 µg/mL IC_50_ value. Acetylcholine is one of the neurotransmitters released by brain cells upon stress [103]. The fruit and bark extract could be a stress-relieving remedy that may help the affected person combat stress.

### 5.14. Miscellaneous

Apart from the pharmacological activities discussed above, *F. religiosa* has been reported for its use as a remedy for polycystic ovary syndrome (PCOS) using an animal model. The female rats were induced with PCOS using letrozole in 0.5% carboxymethyl cellulose (CMC) and orally administered the *F. religiosa* leaves’ (dry and fresh leaves) water extract at the dosage of 1 mg/kg BW for 21 days, consecutively. The findings indicated that treatment using fresh leaf extracts was found to upregulate the *PPAR-γ* and *Cyp19a1* pathways in the ovary to significant levels, similar to the positive control, pioglitazone [104]. These two genes are involved in the insulin-resistance action and the stimulation of androgen production via synthesizing aromatase, respectively. These pathways are involved in the mechanisms of PCOS. The study stated that the water extract also inhibited the synthesis of androgen by reducing the levels of steroidogenic enzymes. The dry leaves’ extract has overall alleviated the steroid imbalances, thus regulate the estrous cycle. The reduction of the multiple ovarian cysts measured by the weight of the ovary was found to be improved by the leaf-treated rats. The study also suggested that the presence of propionic acid and its derivatives, mainly 3-acetoxy 3-hydroxy propionic acid, may have potentially exhibited the activity observed. The compound has previously been reported to improve the PPAR-γ expression, through which PCOS and associated complications are ameliorated [104].

All the pharmacological activities discussed above are summarized in Table 3 below.

## 6. Toxicity Studies

One of the important parts of drug development is toxicological screening, be it for new drugs or for the extension of the existing ones. According to the US Food and Drug Administration (FDA), it is essential to screen any new molecules for their potential therapeutic activity and toxicity potential using animal models. Plants may contain metabolites with synergistic or antagonistic nature, and some may cause serious intoxication or hypersensitivity reactions and, in some cases, may result in anaphylactic shock. Therefore, it is crucial to evaluate the adverse and toxic effects of plant extracts and phytochemical compounds isolated which are intended to be used for human therapy [105].

*F. benghalensis* aerial roots exhibited no signs of toxicity and were considered safe up to 5000 mg/kg in an acute toxicity study. Its crude ethanol root extract was found to be safe up to 3000 mg/kg body weight of Wister albino rats [105]. Meanwhile, the 50% ethanolic leaves’ extract did not cause mortality or any apparent effect on motor activity, feeding behavior, fecal output, or muscular weakness in adult albino mice up to 2000 mg/kg [106]. The albino mice did not exhibit any lethal effect or abnormalities or mortality upon treatment with 2000 mg/kg of the methanolic extract of *F. benghalensis* fruit.

*F. religiosa* leaves’ petroleum ether extract was found to be safe with the absence of mortality up to the dose of 4000 mg/kg when orally administered to adult male Wistar rats [107]. Generally, in toxicity assessment, the median lethal dose (LD_50_) determined is considered poorly toxic when the value is measured to be 2000–5000 mg/kg body weight of the animal model used, and practically non-toxic when it is above 5000 mg/kg. The extracts studied are poorly toxic and are considered for further analyses and application in the pharmaceutical and nutraceutical industry [105].

## 7. Recent Applications

Nanotechnology has recently piqued the interest of researchers due to its effective mechanism. Cellular level penetration and effects of nanoparticles may be beneficial to the pharmacological, nutraceutical, and cosmeceutical industries [108]. The use of plant extracts for the synthesis of nanoparticles is actively performed in recent times. This method is considerably more environmentally friendly and produces more biodegradable waste compared to the routine techniques, which have the opposite effect. 

Sulfur nanoparticles have been reported for their wide uses, especially in agriculture (pesticides and fungicides), medicine (cancer therapy), and catalytic applications (batteries). Synthesis of sulfur nanoparticles using *F. benghalensis* leaves’ extract has been demonstrated by Tripathi et al. [109]. During the procedure, stable nanoparticles’ formation was observed, in which the proteins and phytoconstituents in the leaves act as stabilizing agents, thus enhancing the disproportionation reaction that took place. 

The roots of *F. benghalensis* are also utilized in the green synthesis process as the reducing agent. The water extract of *F. benghalensis* prop root was mixed with silver nitrate solution to obtain silver (Ag_2_O) nanoparticles. The resulting Ag_2_O nanoparticles were examined for their anti-leishmaniasis activity against *Leishmania donovani* [110]. Similarly, another study investigated the antibacterial activity of the Ag_2_O nanoparticles against dental pathogens (*Streptococcus mutans* and *Lactobacilli* sp.) [111]. The nanoparticles analyzed exhibited excellent activity in both studies. 

*F. religiosa* leaves’ aqueous extract was used to synthesize zinc oxide nanoparticles (ZnO) and titanium dioxide nanoparticles (TiO_2_). These nanoparticles showed potential larvicidal activity. The highest mortality rate was observed in the ZnO nanoparticle treatment against fourth-instar *A. stephensi* larvae [112].

Besides the medicinal and nanotechnology applications discussed above, *Ficus* species are also utilized as insecticides and repellent agents. Various medicinal plants are studied for their vector control properties, and these plants have the potential to be developed as environmentally safe vector and pest managing agents. One of the recent studies demonstrated larvicidal effects of *F. religiosa* leaves’ methanolic extract. The extract caused an almost 70% mortality rate in the early third-instar larvae of *Aedes aegypti* [113].

Apart from that, *F. religiosa* leaves’ extracts (ethanol, acetone, benzene, and hexane) displayed significant repellent activity against *C. quinquefasciatus*, *A. stephensi*, and *A. aegypti*, with ethanol extract showing the highest activity at 4.0 mg/cm^2^. These activities are probably due to the presence of terpenoids in the extracts analyzed [114,115]. Both the plant parts were effectively repurposed as insect repellents, thus indicating the vast potential of these species.

## 8. Conclusions and Future Research Prospective

Both the *Ficus* species plant parts have attracted much attention for their various pharmacological potentials contributed by the phytochemicals present in the plant matrix. The species contain a range of flavonoids, phenolics, terpenes and terpenoids, fatty acids, sterols, organic acids, proteins, and some long-chained hydrocarbon compounds. Some of the distinct compounds present in *Ficus* species plant parts include bengalenoside, leucodelphinidin, leucoanthocyanin, leucocyanidin, and derivatives. Besides that, the presence of flavonoids and terpenoids is potentially responsible for their pharmacological activities. 

The studies reviewed confirmed the antimicrobial, antidiabetic, anti-inflammatory, and anticancer activity, which provides a scientific basis for the use of the species in traditional medicines. Moreover, the plants’ constituents are found to be effective mosquito repellents and mosquitocidal agents. However, the effectiveness of the plant parts in urinary and urinogenital disorders of its traditional claim are yet to be discovered scientifically. Comparably, *F. benghalensis* has been investigated more extensively for various pharmacological functions than *F. religiosa*. Some of the potential medicinal actions of *F. religiosa* that need to be explored include anti-stress, antitumor, antiproliferative, antimutagenic, and immunomodulatory actions. The plant may exhibit tremendous activity for its potential phytoconstituents, that mainly consist of flavonoids, phenolics, terpenes, and terpenoids.

Apart from that, bioactivity-based fractionation and bioactive compound isolation should be validated for their abundance, accuracy, reproducibility, and cost-effectiveness. Further analyses should be carried out using animal models to measure the compounds’ potential in the bioactivity observed. These could increase the probability of the optimal use of the phytoconstituents of interest. Overall, the present compilation of chemical constituents with their pharmacological properties will provide prospective information on the existing studies and the research gap or pharmacological aspects that may require further attention and experimental values to be added to this genus. The *Ficus* species can be utilized as functional foods and pharmaceutical ingredients with respect to its pharmacological potentials and its availability in nature. 

## Figures and Tables

**Table 1:** Phytochemical constituents present in *Ficus benghalensis* and *Ficus religiosa* plant parts.

Plant Parts	Compound Class	Compounds Identified	References
*Ficus benghalensis*			
Leaf	Phenolics	Gallic acid, theaflavin-3,3′-digallate, rutin, quercetin-3-galactoside, leucodelphinidin, gallocatechin, kaempferol, apigenin	[2,17]
	Terpenoids/Terpenes	Friedelin, lupeol, β-amyrin, 3-friedelanol, betulinic acid, 20-traxasten-3-ol	
	Miscellaneous	Rhein, anthraquinone, taraxosterol, β-sitosterol, bengalenoside, leucocyanidin, psoralen, bergapten	
Bark	Phenolics	Tannins, leucocyanidin-3-O-β-D-glucopyrancoside, leucopelargonidin-3-O-β-D-glucopyranoside, leucopelargonidin-3-O-α-L-rhamnopyranoside, 5,7-dimethylether-leucopelargonidin-3-0-alpha-L-rhamnoside	[7,18,19,20]
	Terpenoids/Terpenes	Lupeol, lupeol acetate, α-amyrin acetate, gluanol acetate, lanostadienylglucosyl cetoleate	
	Miscellaneous	20-tetratriaconthene-2-one, pentatriacontan-5-one, β-sitosterol, meso-inositol, alpha-D-glucose, beta glucoside, saponin, leucoanthocyanidin, leucoanthocyanin, meso-inositol, bengalensisteroic acid acetate, heneicosanyl oleate, 6-heptatriacontene-10-one, 5,3-dimethyl ether-leucocyanidin-3-0-alpha-D-galactosyl cellobioside	
Aerial root	Terpenoids/Terpenes	Phytol, globulol, lanosterol, lupeol, amyrin acetate, lupenyl acetate, friedelanol, cyclolaudenol, epifriedelanol	[21]
	Miscellaneous	Quinic acid, myristic acid, beta-progesterone, palmitic acid, methyl ester palmitic acid, heptadecanoic acid, linoleic acid, linoleoyl chloride, eicosadienoic acid, methyl ester stearic acid, alpha-monostearin, phthalic acid, dioctyl ester, triacontanol, cycloartanyl acetate, dihydrobrassicasterol, stigmasterol, sitosterol, ergosterol acetate, furostano, 4,22-stigmastadiene-3-one, 1-heptatriacotanol, protodioscin	
Fruit	Miscellaneous	hexadecanoic acid, 5-decenedioic acid and methyl esters of 14,17-octadecadienoic acid, undecanoic acid, 5,6-dimethyl, dimethyl ester, hexadecanoic acid, 14-methyl, hexadecanoic acid,14-methyl, heptadecanoic acid, 16 methyl, oxiraneoctanoic acid, 3 octyl	[22]
*Ficus religiosa*			
Leaf	Phenolics	Eugenol, tannic acid	[23,24]
	Terpenoids/Terpenes	Lupeol, phytol, linalool, α-cadinol, α-eudesmol, β-eudesmol, epi-α-cadinol, γ-eudesmol, epi-γ-eudesmol, α-amyrin	
	Miscellaneous	Campestrol, isofucosterol, *n*-hexadecanoic acid, 12,15-octadecatrienoic acid, octadecanoic acid, butyl-9,12,15-octadecatrienoatet, stigmasterol, *n*-hexanol, adipoin 3-methylcyclopenetane-1,2-dione, phenylacetaldehyde, *n*-nonanal, palmitic acid, pentadecanal, *n*-nonacosane, *n*-hentricontanen, hexa-cosanol, *n*-octacosan	
Bark	Phenolics	Tannin, ceryl behenate, lupeol acetate, α-amyrin acetate, leucopelargonidin-3-O-β-D-glucopyranoside, leucopelargonidin-3-O-α-L-rhamnopyranoside	[8,25]
	Terpenoids/Terpenes	Lanosterol, lupen-3-one	
	Miscellaneous	β-sitosterol, stigmasterol, β-sitosterol-d-glucoside, leucoanthocyanidin, leucoanthocyanin, bergapten, bergaptol	
Stem	Phenolics	2,6-Dimethoxyphenol	[26]
	Miscellaneous	*n*-hexadecanoic acid, octadecanoic acid, stigmasterol, lanosta-8,24-dien-3-ol, acetate(3 beta), ergost-5-en-3-ol(3beta), 4H-Pyran-4-one,2,3-dihydro-3,5-dihydroxy-6-methyl, 2,4-bis(1,1-dimethylethyl)	
Root	Phenolics	Ceryl behenate, lupeol acetate, α-amyrin acetate, leucocyanidin-3-0-β-D-glucopyrancoside, leucopelargonidin-3-0-β-D-glucopyranoside	[27]
	Terpenoids/Terpenes	Lupeol	
	Miscellaneous	Saponin, β-sitosterol, leucoanthocyanidin, leucoanthocyanin	
Fruit	Terpenoids/Terpenes	β-caryophyllene, α-terpinene, dendrolasine, α-trans bergamotene, (e)-β-ocimene, α-pinene, limonene, dendrolasine, α-ylangene, α- thujene, α-copaene, β-bourbonene, aromadendrene, δ-cadinene, α-humulene, β-pinene, alloaromadendrene, germacrene, γ-cadinene, bicyclogermacrene	[28,29]
	Miscellaneous	Stigmasterol, lupeol, undecane, tridecane, tetradecane	

**Table 2:** Microorganisms isolated from *Ficus religiosa* and *Ficus benghalensis*.

*Ficus* Species	Microbial Species	Compound Isolated	Bioactivity/Uses	References
*Ficus religiosa*	*Curvularia lunata*	1-Eicosane	Antimicrobial Cytotoxic properties	[32]
	*Aspergillus* sp.	Naphthaquinone- antibiotic herbarin Herbaridine A	Antidiabetic Anti-inflammatory	[34,35]
*Ficus benghalensis*	*Bacillus subtilis*	Surfactins Iturins	Anti-fungal	[33]

**Table 3:** Pharmacological activities of *Ficus benghalensis* and *Ficus religiosa*.

Species	Bioactivity	Plant Parts	Solvent	Mechanism	Dosage/Concentration/* IC_50_	References
*Ficus benghalensis*	Antioxidants	Seed	Ethanol	DPPH scavenging Nitric oxide Lipid peroxidation FRAP activity	IC_50_ = 446.9 µg/mL IC_50_ = 596.0 µg/mL IC_50_ = 557.0 µg/mL 418.34 µg in 1000 µg AAE	[10]
		Aerial root	Methanol (M) Ethanol (E)	DPPH scavenging FRAP activity	M-IC_50_ = 80.1 µg/mL E- IC_50_ = 38.7 µg/mL M = 982.9 mM Fe^2+^/mg extract E = 261.2 mM Fe^2+^/mg extract	[21]
		Latex	Methanol	DPPH scavenging FRAP activity Phosphomolybdenum activity	IC_50_ = 28.6 µg/mL IC_50_ = 49.8 µg/mL IC_50_ = 31.8 µg/mL	[41]
		Leaves	Hydroethanolic crude (HEC) Hexane fraction (HF)	DPPH scavenging ABTS scavenging	HEC- IC_50_ = 32.3 µg/mL HF- IC_50_ = 28.2 µg/mL HEC- IC_50_ = 52.0 µg/mL HF- IC_50_ = 58.2 µg/mL	[42]
	Antidiabetic	Bark powder	Aqueous	α-glucosidase inhibition Sucrose inhibition	IC_50_ = 77.0 µg/mL IC_50_ = 141.0 µg/mL	[51]
		Leaves	Ethanol	Model: alloxan-induced diabetic albino rats Duration: 14 days Route: Oral administration Action: reduced the triglycerides, cholesterol, and glucose	200 mg/kg (BW) ** 400 mg/kg (BW)	[52]
		Bark	Ethanol	Model: STZ-induced diabetic rats Route: Oral administration Duration: 15 days Action: significant reduction in blood glucose levels stimulation of insulin secretion from beta cells of Islets of Langerhans	150 mg/kg (BW) 300 mg/kg (BW) 500 mg/kg (BW)	[53]
	Anti-inflammatory	Root	Ethanol	Model: Excision and incision wound Route: Topical administration Duration: 10 days Action: accelerated wound healing decreased epithelization period increased wound contraction rate increased the closure of the excision, enhancing the epithelization increased the granuloma tissue-breaking strength	200 mg/kg (BW)	[63]
		Bark	Aqueous Ethanol	Model: Freund’s complete adjuvant-induced arthritis rat Route: Oral administration Duration: 21 days Action: normalized body weight, arthritic score, ankle diameter, and paw volume protective effects on the primary and secondary lesions	200–400 mg/kg (BW)	[24]
		Bark	Aqueous (A) Ethanol (E)	Model: Incision wound Route: Oral administration Duration: 10 days Action: significant increase in the wound-breaking strength	200 mg/kg (BW) Wound breaking strength: A = 447.7 g; E = 466.7 g	[62]
		Bark	Ethanol Aqueous	Model: Excision wound Route: Topical administration Duration: 20 days Action: accelerated wound healing, decreased epithelization period significant reduction in the wound area increased wound contraction rate increased the closure of the excision, enhancing the epithelization increased the granuloma tissue-breaking strength	10% ointment formulation	[62]
		Leaves	50% Ethanol	Model 1: Pylorus ligation + aspirin-induced gastric ulcer Route: Oral administration Duration: 3 days Action: reduction of damage in the mucosa Ulcer indices (mm^2^/rat) were reduced, and protection percentage (%) increased in a dose-dependent manner	100 mg/kg (BW) (29.2 mm^2^/35.8%) 200 mg/kg (BW) (22.2 mm^2^/56.2%) 400 mg/kg (BW) (12.2 mm^2^/77.7%)	[64]
				Model 2: Acetic acid-induced gastric ulcer Route: Oral administration Duration: 10 days Action: reduction of damage in the mucosa Ulcer indices (mm^2^/rat) were reduced, and protection percentage (%) increased in dose-dependent manner	100 mg/kg (BW) (10.7 mm^2^/31.6%) 200 mg/kg (BW) (6.3 mm^2^/12.0%) 400 mg/kg (BW) (2.2 mm^2^/6.7%)	
				Model 3: Ethanol-hydrochloric acid-induced gastric ulcer Route: Oral administration Duration: 8 days Action: reduction of damage in the mucosa Ulcer indices (mm^2^/rat) were reduced, and protection percentage (%) increased in a dose-dependent manner	100 mg/kg (BW) (14.7 mm^2^/41.6%) 200 mg/kg (BW) (12.3 mm^2^/62.0%) 400 mg/kg (BW) (8.2 mm^2^/80.7%)	
	Analgesic	Root	Aqueous	Model: Swiss albino mice Hot-plate reaction time (13.6 s; 10.3 s) Tail-flick reaction time (13.6 s; 10.3 s) Writhing test (36.0 in 10 min)	100 mg/kg (BW) 200 mg/kg (BW)	[66]
	Antiproliferative	Stem bark	Butanol fraction	Cell: Yeast cells Assay: *Allium cepa* root tip assay Yeast cell model Action: caused chromosomal and mitotic aberrations (accumulation of prophases, sticky chromosomes at metaphase, spindle disturbance at prophase, and anaphase bridges)	Cell viability = 8% at 4 mg/mL Mitotic index = 96% at 4 mg/mL	[70]
	Anticancer	Aerial roots	Ethyl acetate	Cell lines: Lung cancer (A549) Breast cancer (MDA-MB-231) Cervical cancer (Hela)	Cell viability: IC_50_ = 17.8 µg/mL IC_50_ = 97.9 µg/mL IC_50_ = 49.3 µg/mL	[73]
		Leaves (L) Branch (B)	Methanol	Cell lines: Hepatocellular carcinoma (HepG2) Breast cancer (MCF-7)	Cell viability at 100 ppm HepG2: L = 22.1% B = 8.3% MCF-7: L = 16.4% B = 14.8%	[75]
	Antimutagenic	Stem bark	Aqueous	Bacteria strain: *Salmonella typhimurium* TA100	IC_50_ = 70.24 mg/mL	[40]
	Antimicrobial	Root	Ethanol	Bacteria strains/ Inhibition zone: *Staphylococcus aureus* Inhibition zone: 20 mm, 25 mm, 30 mm *Escherichia coli* Inhibition zone: 15 mm, 20 mm, 24 mm *Klebsiella pneumonia* Inhibition zone: 12 mm, 18 mm, 22 mm	25 mg/mL 50 mg/mL 75 mg/mL	[19]
		Aerial root	Methanol Ethanol	Bacteria strains/ Inhibition zone: *Vibrio anguillarum* and *Enterococcus faecalis* Inhibition zone > 20 mm	200, 100, 50, 25, 12.5, and 6.25 μg per disc	[21]
		Aerial root (AR) Leaves (L) Fruit (F)	Ethanol	Bacteria strains/Inhibition zone (AR/L/F): *Staphylococcus aureus*: 0.4, 0.6, 0.7 mm/0.6, 0.7, 0.9 mm/0.6, 0.7, 0.9 mm *Escherichia coli*: 0.6, 0.7, 0.8 mm/0.6, 0.8, 0.9 mm/0.6, 0.8, 0.9 mm *Pseudomonas protobacteria*: 0.3, 0.4, 0.5 mm/0.3, 0.4, 0.5 mm/0.3, 0.4, 0.5 mm *Bacillus cereus*: 0.4, 0.6, 0.7 mm/0.5, 0.6, 0.7 mm/0.5, 0.6, 0.7 mm Action: moderate activity	50 µL 100 µL 150 µL	[81]
		Leaves	Aqueous	Viral strains: HIV-1UG070 and HIV-1VB59 in TZM-bl and PM1 cells Action: moderate activity	HIV-1UG070: IC_80_ = 6 μg/mL; 6 μg/mL HIV-1VB59: IC_80_ = 5.2 μg/mL; 2.25 μg/mL	[82]
	Antihelminth	Fruit	Aqueous	Earthworm: *Pheretima* *Posthuma* Duration: 3 h Action: 100% mortality within 1 h of exposure	37.5 mg/mL	[87]
		Latex	-	Earthworm: *Pheretima* *Posthuma* Duration: 1 h Action: Caused paralysis and eventually death	250 μL 500 μL	[87]
	Hepatoprotective	Latex	Methanol	Model: CCl_4_-induced hepatotoxicity in albino rats Route: Oral administration Duration: 21 days Action: decreased SGPT, SGOT, ALP, and bilirubin level increased total protein level	300 mg/kg (BW)	[92]
	Anticoagulant	Leaves	Methanol	Prothrombin time (PT) 17.7 to 26.7 s Activated partial thromboplastin time (APTT) 47.7 to 72.3 s		[99]
	Immunomodulatory	Leaves	Butanol fraction	Action: Induced phagocytosis in *Candida albicans*	1000 mg/mL	[42]
*Ficus religiosa*	Antioxidant	Stem bark	Crude methanol, n-hexane, dichloromethane, ethyl acetate fractions	DPPH scavenging	>90% inhibition	[44]
		Bark	Ethanol (E) Methanol (M)	DPPH scavenging	E = 70.5% M = 69.0%	[45]
			Methanol	DPPH scavenging	IC_50_ = 48 μg/mL	[46]
		Leaves	Methanol	H_2_O_2_ scavenging	IC_50_ = 49.85 µg/mL	[47]
	Antidiabetic	Bark	Aqueous	Model: STZ-induced diabetic Route: Oral administration Duration: 21 days Action: reduced hyperglycemia increased insulin and glycogen uptake in liver and skeletal muscle reduced serum triglyceride and total cholesterol reduced lipid peroxidation	250 mg/kg (BW)	[55]
			Methanol	α-glucosidase inhibition	IC_50_ = 83.72 μg/mL	[56]
	Anti-inflammatory	Latex	Methanol	Model: Cisplatin-induced liver injury Route: Oral administration Duration: 16 days Action: reduction in the hyaline droplets, tubular dilation, and recovery normalized serum urea and creatinine reduced lipid oxidation normalized renal biomarkers (glutathione (GSH), superoxide dismutase (SOD), catalase (CAT), ATPase (Na^+^/K^+^, Ca^2+^, and Mg^2+^))	200 mg/kg (BW) 300 mg/kg (BW)	[67]
		Bark Leaves aerial roots	Aqueous	cell culture real-time polymerase chain reaction (PCR) downregulation of metalloproteinase-1 (*MMP*) matrix astringent activity wound healing assay, enhanced wound healing through re-epithelization	0.02, 0.05, 0.1, and 0.5 mg/mL	[46]
		Bark	Extract and ash-methanolic chloroform	Model: Burned wound Sprague Dawley Route: Topical application Duration: 15 days Action: 100% wound contraction lower wound closure time progressive re-epithelization formation of granulation tissues cellular proliferation	10% extract Formulation	[68]
		Leaves	Methanol	Model: Ethanol-induced gastric lesions Route: Oral administration Duration: 2 h Action: reduce ulcer index through the action against the 5-lipoxygenase pathway stimulates the prostaglandin synthesis, protects the gastric mucosa	250 mg/kg (BW) 500 mg/kg (BW)	[14]
				Model: Aspirin-induced gastric ulcer Route: Oral administration Duration: 10 days Action: reduce ulcer index through the action against the 5-lipoxygenase pathway and inhibition of leukotrienes’ production stimulates the prostaglandin synthesis, protects the gastric mucosa	250 mg/kg (BW) 500 mg/kg (BW)	
				Model: Pylorus ligated rats Route: Oral administration Duration: 7 days Action: reduced ulcer index stimulates the prostaglandin synthesis, protects the gastric mucosa	250 mg/kg (BW) 500 mg/kg (BW)	
	Anticancer	Bark	Aqueous	Anti-neoplastic activity Cells: cervical cancer cell lines (SiHa-HPV16-positive and HeLa-HPV18-positive) Action: reduced the growth of cancer cell upregulated the expression of *p53*, *p21*, and *pRb* proteins downregulates the phospho Rb (*ppRb*) protein expression terminates the cell cycle progression at the G1/S phase in SiHa Induces apoptosis in HeLa (increasing the intracellular Ca^2+^ level, resulting in the loss of mitochondrial membrane potential) promotes the release of cytochrome-c, upregulated the caspase-3 expression downregulates *MMP-2* and *Her-G2* downregulates viral oncoproteins *E6* and *E7* expression	0–80 µg/mL	[74]
		Leaves	Benzene (B) Acetone (A)	Cell: breast cancer cells (MCF-7) MTT assay Action: inhibit cell growth	Cell viability (IC_50_) B = 160.3 μM A = 222.7 μM	[76]
		Latex	Ethanol	Model: human neuroblastoma IMR 32 (cell inhibition: 4.8 μg/mL), human colorectal HCT 116, and human breast adenocarcinoma MDA MB 231 Action: cell arrest and accumulation at G1 phase (HCT 116 and MDA MB 231) cell arrest and accumulation at G2/M phase (*IMR 32*) induced apoptosis upregulation of pro-apoptotic (*caspase-3* and *p53*) downregulation of anti-apoptotic (*Bcl-2, AKT*) genes		[77]
		Bark	Methanol	Model: human breast adenocarcinoma Action: maximum cell death stimulated early apoptosis and apoptosis (86.3% apoptotic cells in the G0/G1 population) upregulation of *BAX* and proteolytic cleavage of *PARP-1* downregulated *Bcl-2* genes	91 µg/mL	[78]
	Antimicrobial	Fruit	Ethanol	Bacteria strains/ Inhibition zone: *K. pneumonia*: 21 mm *S. epidermidis*: 19 mm	15 mg/mL	[80]
		Stem	Ethanol	Fungal strain: *Candida albicans*: 10.6 mm	5 mL extract solution	[85]
	Antihelminthic	Latex	-	Earthworm: *Pheretima* *posthuma* Duration: 3 h Action: Paralysis in the earthworms and causes death	250 µL 500 µL	[90]
	
	Hepatoprotective	Stem bark	Ethanol	Model: CCl_4_-induced hepatotoxicity in albino rats and paracetamol-induced hepatic damage in rats Route: Oral administration Duration: 36 h Action: reduction in the serum aspartate aminotransferase (AST) and alanine aminotransferase (ALT) reduced the liver tissue injury reduced the negative effects caused by paracetamol metabolites and CCL_3_ radical	200 mg/kg (BW)	[95]
	Anticoagulant	Leaves	Methanol	Prothrombin time (PT): 17.7 to 26.7 s Activated partial thromboplastin time (APTT): 47.7 to 72.3 s	1 μg/μL	[99]
	Fertility	Leaves (fresh and dry)	Aqueous	Model: Letrozole-induced PCOS Route: Oral administration Duration: 21 days Action: upregulate the *PPAR-γ* and *Cyp19a1* pathways in the ovary insulin resistance action stimulation of androgen production via synthesizing aromatase alleviated the steroid imbalances, thus regulating the estrous cycle reduction of the multiple ovarian cysts	1 mg/kg (BW)	[104]
*Ficus* *benghalensis & Ficus religiosa*	Antioxidant	Leaves	Methanol	DPPH scavenging H_2_O_2_ scavenging	Inhibition percentage > 80% at 100 µg/mL IC_50_ = 49.85 µg/mL	[47]
	Cell proliferation activity	Leaves	Methanol	Cell line: Human cervical cancer cell line (HeLa) Assay: Mitochondrial reduction assay	Cell viability: 50% at 100 µg/mL	[47]

Note: * IC_50_ = half maximal inhibition constant; ** BW = body weight.
